# A Novel Modified Hydrated Sodium Calcium Aluminosilicate (HSCAS) Adsorbent Can Effectively Reduce T-2 Toxin-Induced Toxicity in Growth Performance, Nutrient Digestibility, Serum Biochemistry, and Small Intestinal Morphology in Chicks

**DOI:** 10.3390/toxins11040199

**Published:** 2019-04-02

**Authors:** Jin-Tao Wei, Kun-Tan Wu, Hua Sun, Mahmoud Mohamed Khalil, Jie-Fan Dai, Ying Liu, Qiang Liu, Ni-Ya Zhang, De-Sheng Qi, Lv-Hui Sun

**Affiliations:** 1 Department of Animal Nutrition and Feed Science, College of Animal Science and Technology, Huazhong Agricultural University, Wuhan 430070, China; jintao001@163.com (J.-T.W.); kuntanwu@webmail.hzau.edu.cn (K.-T.W.); huasun@webmail.hzau.edu.cn (H.S.); zhangniya@mail.hzau.edu.cn (N.-Y.Z.); qds@mail.hzau.edu.cn (D.-S.Q.); 2 Key Laboratory of Animal Embryo Engineering and Molecular Breeding of Hubei Province, Institute of Animal Husbandry and Veterinary Sciences, Hubei Academy of Agricultural Sciences, Wuhan 430064, China; 3 Animal Production Department, Faculty of Agriculture, Benha University, Benha 13736, Egypt; mahmoud.khalil@fagr.bu.edu.eg; 4 Sichuan Green Food Development Center, Chengdu 610041, China; daijiefan@126.com; 5 Tianjin Animal Disease Prevention and Control Center, Tianjin 300402, China; yingliuadpcc@sohu.com; 6 Jiangsu Aomai Bio-Technology Co., Ltd., Nanjing 211226, China; liuayang@njau.edu.cn

**Keywords:** modified HSCAS, absorption, T-2 toxin, broilers

## Abstract

The objective of this study was to evaluate the ability of a modified hydrated sodium calcium aluminosilicate (HSCAS) adsorbent to reduce the toxicity of T-2 toxin in broilers. Ninety-six one-day-old male broilers were randomly allocated into four experimental groups with four replicates of six birds each. The four groups, 1–4, received a basal diet (BD), a BD plus 6.0 mg/kg T-2 toxin, a BD plus 6.0 mg/kg T-2 toxin with 0.05% modified HSCAS adsorbent, and a BD plus 0.05% modified HSCAS adsorbent, respectively, for two weeks. Growth performance, nutrient digestibility, serum biochemistry, and small intestinal histopathology were analyzed. Compared to the control group, dietary supplementation of T-2 toxin decreased (*p* < 0.05) body weight gain, feed intake, and the feed conversion ratio by 11.4–31.8% during the whole experiment. It also decreased (*p* < 0.05) the apparent metabolic rates of crude protein, calcium, and total phosphorus by 14.9–16.1%. The alterations induced by T-2 toxin were mitigated (*p* < 0.05) by the supplementation of the modified HSCAS adsorbent. Meanwhile, dietary modified HSCAS adsorbent supplementation prevented (*p* < 0.05) increased serum aspartate aminotransferase by T-2 toxin at d 14. It also prevented (*p* < 0.05) T-2 toxin-induced morphological changes and damage in the duodenum, jejunum, and ileum of broilers. However, dietary supplementation of the modified HSCAS adsorbent alone did not affect (*p* > 0.05) any of these variables. In conclusion, these findings indicate that the modified HSCAS adsorbent could be used against T-2 toxin-induced toxicity in growth performance, nutrient digestibility, and hepatic and small intestinal injuries in chicks.

## 1. Introduction

Trichothecenes are secondary fungal metabolites largely produced by *Fusarium, Trichoderma*, and *Mycothecium* species [1]. T-2 toxin has shown the highest toxicity of the commonly tested type A trichothecenes [1]. T-2 toxin has been detected in grains and animal feed all over the world [2,3]. Previous reports have shown that about 20–70% of European cereal samples, including maize, barley, and wheat, have T-2 toxin [2,3]. T-2 toxin can be quickly absorbed in the intestinal tract, and then causes severe damage to various organs of animals, especially the liver and the digestive system [4,5]. After consumption, T-2 toxin is known to reduce feed intake and weight gain in mice [6], broiler chickens [7], and pigs [8]. Furthermore, many studies have considered T-2 toxin impacts on the relative weight of organs [9], serum biochemistry [10], restrained protein synthesis [1], cell apoptosis [11], and the suppression of immune functions [1]. Therefore, the development of effective strategies to reduce T-2 toxicity has attracted much interest over the past few decades. 

Generally, there are several methods to reduce the harmful effects of T-2 toxin, including physical, chemical, and biological procedures. Irradiation provides intense energy to break down T-2 toxin in grains [12], and strong alkaline solutions can inhibit T-2 toxin biological activity [13]. Furthermore, enzymatic treatment can also degrade T-2 toxin, destroying its 12,13-epoxide ring [14]. However, methods to remove T-2 toxin from feed and food can be unstable and expensive and can further affect grain quality [15]. Physical adsorption is more effective and directly detoxifies mycotoxins by inhibiting absorption in the gastrointestinal tract [16], but there is a lack of efficient adsorbent for T-2 toxin and deoxynivalenol (DON) [17]. Previous studies have reported that adsorbents contain aluminosilicates, such as bentonite [18], montmorillonite [19], and zeolite [20], displaying an ability to effectively protect against zearalenone [21], aflatoxin B_1_ (AFB_1_), and fumonisin B_1_ (FB_1_) [22] in several farm and experimental animals. Hydrated sodium calcium aluminosilicate (HSCAS) is a material obtained by using natural zeolite ore: It is purified through crushing, screening, and high-temperature treatment, allowing the structure to increase its size, surface area, and adsorption volume [23]. Amdetox^TM^ is an adsorbent that mainly contains HSCAS whose surface is modified by cetylpyridinium chloride and the intercalation of β-glucan. The modified HSCAS adsorbent increases surface area and might be able to increase adsorbing mycotoxins and avoid adsorbing the nutrients in feed. The objective of this study was to determine the ameliorative effects of Amdetox^TM^ detoxification on the toxicity induced by T-2 toxin. 

## 2. Results

### 2.1. Growth Performance

Growth performance results are presented in Table 1. Nonsignificant differences in initial body weight were observed among the four groups (*p* > 0.05, Table 2). Compared to the control (group 1), dietary T-2 supplementation (group 2) decreased (*p* < 0.05) body weight gain and feed intake by 15.3–31.8% and 12.4–20.6%, respectively, during d 1–7, d 8–14, and d 1–14, while it increased (*p* < 0.05) feed intake by 11.4–15.9% during d 8–14 and d 1–14. Although dietary supplementation of Amdetox^TM^ (group 3) did not alleviate T-2 toxin-induced (group 2) adverse effects on body weight gain and feed intake in d 1–7, dietary supplementation of Amdetox^TM^ (group 3) alleviated T-2 toxin-induced (group 2) adverse effects on body weight gain and feed intake by 38.6–46.6% and 33.0–36.0%, respectively, during d 8–14 and d 1–14. Meanwhile, dietary supplementation of Amdetox^TM^ (group 3) mitigated the reduced feed/gain induced by T-2 toxin (group 2) throughout the experiment. Notably, dietary-supplemented Amdetox^TM^ alone (group 4) did not affect (*p* > 0.05) body weight gain, feed intake, and feed/gain compared to the control (group 1) throughout the experiment. Notably, the variation in growth performance was very low for each treatment, indicating that in the current study, the results from four replicates per treatment might be reliable. 

### 2.2. Apparent Metabolic Rate

The nutrient metabolic rate results are shown in Table 2. Although dietary supplementation of T-2 toxin (group 2) did not affect (*p* > 0.05) the apparent metabolic rate of gross energy, it decreased (*p* < 0.05) the apparent metabolic rate of crude protein, calcium, and total phosphorus by 14.9%, 18.0%, and 16.1%, respectively. Notably, the changes in the apparent metabolic rates of crude protein and total phosphorus induced by T-2 toxin were alleviated by dietary supplementation of Amdetox^TM^ (group 3) when compared to the control (group 1). In addition, dietary-supplemented Amdetox^TM^ alone (group 4) did not affect (*p* > 0.05) the apparent metabolic rates of gross energy, crude protein, calcium, and total phosphorus compared to the control (group 1).

### 2.3. Serum Biochemistry and Histopathology

**Figure 1:**
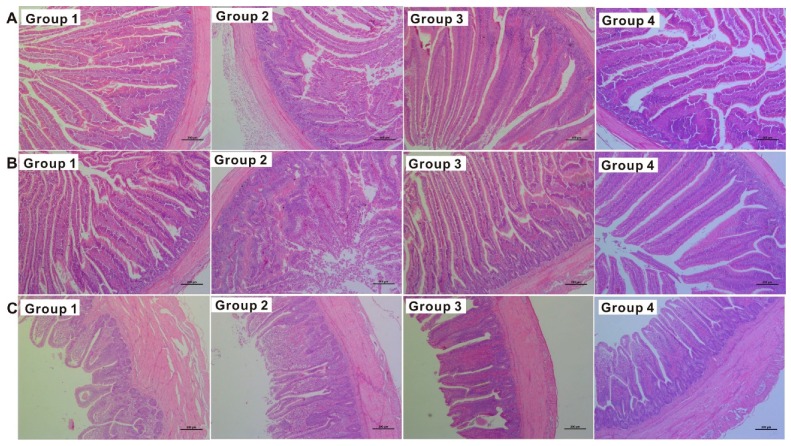
Effects of dietary T-2 toxin and Amdetox^TM^ on histopathology of the (**A**) duodenum, (**B**) jejunum, and (**C**) ileum of chicks. The sections were stained with hematoxylin and eosin. Photomicrographs are shown at 100× magnification. Group 1 = basal diet; group 2 = basal diet + 6 mg/kg T-2 toxin; group 3 = basal diet + 6 mg/kg T-2 toxin + 0.05% of Amdetox^TM^; group 4 = basal diet + 0.05% of Amdetox^TM^.

Serum biochemistry results are presented in Table 3. After two weeks of experimental treatments, although T-2 toxin (group 2) did not affect (*p* > 0.05) serum alanine aminotransferase (ALT), total protein (TP), and albumin (ALB), it increased (*p* < 0.05) aspartate aminotransferase (AST) (17.7%) relative to the control (group 1). Strikingly, this change was inhibited by dietary supplementation of Amdetox^TM^ (group 3). Dietary-supplemented Amdetox^TM^ alone (group 4) did not affect (*p* > 0.05) these serum biochemistry variables compared to the control (group 1). Additionally, the histological results showed that dietary T-2 toxin supplementation induced intestinal injury (Figure 1). Specifically, compared to the control (group 1), T-2 toxin (group 2) induced severe degeneration, necrosis, and desquamation of the villous epithelial cells; and increased inflammatory cells in the intestinal mucosa, congestion in the intestinal lamina propria, goblet cell hyperplasia in the intestinal gland, and/or edema and thickening in the serosa, with an infiltration of numerous lymphoid cells in the duodenum (Figure 1A), jejunum (Figure 1B), and ileum (Figure 1C). Intriguingly, dietary supplementation of Amdetox^TM^ (group 3) prevented T-2 toxin-induced (group 2) injury in the small intestine. In contrast, intestinal morphology was not affected by the supplementation of Amdetox^TM^ alone (group 4). 

## 3. Discussion

The current study demonstrates that the modified HSCAS adsorbent could effectively counteract T-2 toxin-induced adverse effects on broilers. Chick consumption of T-2 toxin reduced body weight, feed intake, and feed conversion, which was in accordance with previous studies [7,24]. The poor growth performance of broilers, induced by T-2 toxin, was further proven to be associated with decreased metabolic rates of crude protein, calcium, and total phosphate in chicks in the current study. These outcomes are similar to previous studies that showed that *Fusarium* toxins can negatively affect nutrient digestibility in chicks [24,25]. Interestingly, the current study showed that dietary supplementation of 0.05% Amdetox^TM^ successfully reduced the negative effect induced by T-2 toxin. Notably, no negative effects in these productive parameters were found between broilers in the experimental group supplemented with Amdetox^TM^ alone and the control group, indicating that Amdetox^TM^ was nontoxic and safe.

The small intestine, including the duodenum, jejunum, and ileum, is the major part where most nutrient digestion and absorption takes place [26]. In this study, the pathological results showed that T-2 toxin caused serious small intestinal detriment in broilers, and these outcomes are in agreement with previous reports that T-2 toxin could induce intestine damage, thus decreasing nutrient utilization efficiency, as described above [27]. Interestingly, T-2 toxin-induced injury in the small intestine was mitigated by Amdetox^TM^ supplementation. Furthermore, the administration of T-2 toxin alone increased AST activity compared to the control group. The activity of serum enzymes such as AST and ALT, and concentrations of serum ALB and TP, have been described as valuable parameters of hepatic function and injury [28]. These outcomes are similar to previous studies that provided evidence that liver injury was induced by T-2 toxins in chicks [29,30]. However, some other reports have shown that T-2 toxin did not affect these parameters in poultry [31], pigs [32], and hamsters [33]. This discrepancy could be attributed to different experimental conditions, including exposure doses, duration, and animal species. The results obtained from the current study show that serum biochemical changes could be ameliorated by Amdetox^TM^ supplementation. Taken together, these results are in agreement with previous studies that reported that growth retardation induced by T-2 toxin was mainly due to induced intestinal and hepatic injury. Dietary Amdetox^TM^ supplementation, however, prevented T-2 toxin-induced poor growth performance, which was associated with the inhibition of intestinal and hepatic injury.

HSCAS adsorbent is a commercial feed additive that has been proven to have an effective ability to adsorb AFB_1_ [34,35], while several studies have reported that general HSCAS adsorbents could not effectively adsorb Trichothecenes such as T-2 toxin [36] and DON [37]. Interestingly, artificially modified substances of zeolites [38], glucomannan [39], montmorillonite [40], and diatomaceous earth [30] can enhance the adsorption and detoxification of mycotoxins. Therefore, a modified HSCAS adsorbent product, Amdetox^TM^, was developed to prevent the harmful effects of T-2 toxin. This HSCAS adsorbent is surface-modified with cetylpyridinium chloride based on natural bentonite and is intercalated with yeast β-glucan. After the special bentonite interlayer cation and water molecules are replaced by modifiers, the spacing between the particles is significantly increased, and the surface of the particles changes from hydrophilic to hydrophobic. Therefore, the modified HSCAS adsorbent has a larger adsorption capacity and lipophilic hydrophobicity, and it could effectively adsorb various mycotoxins in the feed. As expected, the modified HSCAS adsorbent displayed an effective ability to prevent the negative effect of T-2 toxin in broilers. Previous studies have shown that 0.25% adsorbent or 2% polymeric glucomannans could alleviate the harmful effect of 1.0–2.0 mg/kg T-2 toxin [41,42]. An addition of only 0.05% Amdetox^TM^ to a diet could reduce the negative effect induced by 6.0 mg/kg T-2 toxin, indicating that the modified HSCAS adsorbent was quite effective. Meanwhile, consistent with previous studies [19,43], the modified HSCAS adsorbent did not affect nutrient metabolic rates and growth performance in the current study.

## 4. Conclusions

In summary, these data indicate that the modified HSCAS adsorbent, Amdetox^TM^, could be used as a promising adsorbent for the detoxification of T-2 toxin in practice.

## 5. Materials and Methods 

### 5.1. Birds, Treatment, and Growth Performance

Ninety-six one-day-old Cobb-500 male broiler chicks with similar body weights were randomly allocated into four groups with four replicates of six birds/cage. The four groups of birds were allowed free access to water and were fed a corn-soybean-based diet (BD, group 1) formulated to meet the nutritional requirements of broilers (National Research Council (NRC), 1994; Table 4) or BD supplemented with 6.0 mg/kg T-2 toxin (Pribolab Pte. Ltd., Singapore) (group 2), 6.0 mg/kg T-2 toxin with 0.05% of Amdetox^TM^ (Jiangsu Aomai Bio-technology Co., Ltd. Nanjing, China) (group 3), or 0.05% of Amdetox^TM^ (group 4). The T-2 toxin-contaminated diet was made through a stepwise dilution method. Briefly, 150 mg of T-2 toxin was dissolved in 50 mL of ethanol and then mixed with 500 g of corn. The mixed sample was subsequently dried at 65 °C in an oven, which was used to make the T-2 toxin-contaminated diets. The mortality of the birds was monitored daily, whereas feed intake and body weight were measured weekly. The total excreta of each pen were collected during d 8–14 to measure the apparent metabolic rates of gross energy, crude protein, calcium, and total phosphorus of chicks. The experiment lasted for two weeks. At the end of the experiment, eight birds from each treatment group were killed to collect blood and small intestine (duodenum, jejunum, and ileum) for serological and intestinal histological examinations, as previously described [44]. 

### 5.2. Serum Biochemistry and Histopathology

The serum was prepared by centrifugation of the blood at 1000× *g* at 4 °C for 10 min. The activities of ALT and AST, as well as the concentrations of TP and ALB, in the serum were measured with the use of an automatic biochemistry analyzer (Beckman Synchron CX4 PRO, CA, USA), as previously described [45]. The duodenum, jejunum, and ileum (*n* = 4/group) were removed, fixed in neutral-buffered 10% formalin, embedded in paraffin, sectioned at 5 μm, stained with hematoxylin and eosin (H&E), and examined microscopically for histopathology [46]. Briefly, intestinal tissue was examined for each chick by light microscopy for described lesions: Degeneration, necrosis, and desquamation of the villous epithelial cells; and edema, thickening in the serosa, or both. Sections with no, slight, moderate, or intense presence of lesions were given scores of 0, 1, 2, and 3, respectively [46].

### 5.3. Apparent Metabolic Rate

The apparent metabolic rates of gross energy, crude protein, calcium, and total phosphorus of chicks were measured and calculated as previously described [47]. Gross energy was analyzed using an adiabatic bomb calorimeter standardized (IKA C2000) with benzoic acid. Calcium, total phosphorus, and crude protein were measured following the permanganate titration method 990.03 (AOAC, 2000), the colorimetric determination method 985.01 (AOAC, 1990), and the Kjeldahl digestion method 984.13 (AOAC, 1990), respectively [48]. 

### 5.4. Statistical Analysis

A one-way ANOVA was used to test the main effects of the dietary effect. A Bonferroni *t*-test followed for multiple mean comparisons if there was a main effect. Data are presented as means ± SD, and the significance level was set at *p* < 0.05. The analyses were conducted using the SPSS Statistics 19.0 package (SPSS Inc., IBM, New York, Ny, USA).

### 5.5. Ethical

This research was approved by Scientific Ethic Committee of Huazhong Agricultural University on 20 March 2017. The project identification code is HZAUCH-2017-008.

## Figures and Tables

**Table 1:** Effects of T-2 toxin and Amdetox^TM^ on growth performance of broilers. ^1^

Item	Group 1	Group 2	Group 3	Group 4
Initial body weight, g/bird	54.3 ± 0.4	54.4 ± 0.7	54.5 ± 0.7	54.0 ± 0.2
d 1 to 7				
Body weight gain, g/bird	138.9 ± 6.2 ^a^	117.6 ± 5.4 ^b^	115.9 ± 8.7 ^b^	136.2 ± 18.4 ^a^
Feed intake, g/bird	167.6 ± 3.8 ^a^	146.9 ± 4.8 ^b^	149.1 ± 5.3 ^b^	166.3 ± 1.8 ^a^
Feed/gain, g/g	1.21 ± 0.05	1.25 ± 0.08	1.29 ± 0.06	1.24 ± 0.17
d 8 to 14				
Body weight gain, g/bird	280.2 ± 17.0 ^a^	191.1 ± 10.1 ^c^	232.6 ± 11.9 ^b^	289.8 ± 22.7 ^a^
Feed intake, g/bird	385.2 ± 11.9 ^a^	305.8 ± 10.7 ^c^	334.4 ± 28.9 ^b^	376.3 ± 18.8 ^a^
Feed/gain, g/g	1.38 ± 0.05 ^b^	1.60 ± 0.07 ^a^	1.44 ± 0.05 ^b^	1.30 ± 0.06 ^b^
d 1 to 14				
Body weight gain, g/bird	419.2 ± 21.7 ^a^	308.7 ± 8.1 ^c^	351.3 ± 11.6 ^b^	426.0 ± 9.1 ^a^
Feed intake, g/bird	552.8 ± 11.9 ^a^	452.7 ± 14.5 ^b^	485.7 ± 30.7 ^b^	542.9 ± 17.9 ^a^
Feed/gain, g/g	1.32 ± 0.05 ^b^	1.47 ± 0.02 ^a^	1.38 ± 0.05 ^b^	1.27 ± 0.02 ^b^

^a–c^ Means within a row lacking a common superscript differ significantly (*p <* 0.05). ^1^ Results are reported as the mean ± SD, *n* = 4. Group 1 = basal diet; group 2 = basal diet + 6 mg/kg T-2 toxin; group 3 = basal diet + 6 mg/kg T-2 toxin + 0.05% of Amdetox^TM^; group 4 = basal diet + 0.05% of Amdetox^TM^.

**Table 2:** Effects of T-2 toxin and Amdetox^TM^ on the metabolic rates of gross energy, crude protein, calcium, and total phosphorus of broilers during d 8–14. ^1^

Item	Group 1	Group 2	Group 3	Group 4
Gross energy, %	67.1 ± 3.0	64.1 ± 2.8	67.0 ± 3.6	66.1 ± 2.6
Crude protein, %	57.6 ± 2.5 ^a^	49.0 ± 5.0 ^b^	52.5 ± 6.9 ^ab^	55.3 ± 1.7 ^a^
Calcium, %	40.5 ± 5.2 ^a^	33.2 ± 4.9 ^b^	30.6 ± 1.1 ^b^	34.9 ± 10.9 ^ab^
Total phosphorus, %	52.7 ± 6.9 ^a^	44.2 ± 4.8 ^b^	48.4 ± 8.1 ^ab^	49.3 ± 7.8 ^ab^

^a–b^ Means within a row lacking a common superscript differ significantly (*p <* 0.05). ^1^ Results are reported as the mean ± SD, *n* = 4. Group 1 = basal diet; group 2 = basal diet + 6 mg/kg T-2 toxin; group 3 = basal diet + 6 mg/kg T-2 toxin + 0.05% of Amdetox^TM^; group 4 = basal diet + 0.05% of Amdetox^TM^.

**Table 3:** Effects of T-2 toxin and Amdetox^TM^ on serum biochemistry of broilers at d 14. ^1^

Item	Group 1	Group 2	Group 3	Group 4
ALT/(U/L)	2.68 ± 0.12	2.85 ± 0.34	2.47 ± 0.27	2.80 ± 0.19
AST/(U/L)	196.7 ± 6.0 ^b^	231.5 ± 5.2 ^a^	203.0 ± 3.4 ^b^	200.6 ± 7.9 ^b^
TP/(g/L)	25.7 ± 1.6	27.2 ± 1.2	28.4 ± 1.0	27.4 ± 1.9
ALB/(g/L)	12.7 ± 1.0	13.1 ± 0.8	13.3 ± 0.6	13.7 ± 1.3

^a–b^ Means within a row lacking a common superscript differ significantly (*p* < 0.05). ^1^ Results are reported as the mean ± SD, *n* = 8. ALT = alanine transaminase; AST = aspartate aminotransferase; TP = total protein; ALB = albumin. Group 1 = basal diet; group 2 = basal diet + 6 mg/kg T-2 toxin; group 3 = basal diet + 6 mg/kg T-2 toxin + 0.05% of Amdetox^TM^; group 4 = basal diet + 0.05% of Amdetox^TM^.

**Table 4:** Formulation and nutritional content of the basal diet. ^1^

Ingredients (%)	Quantity (%)
Corn	54.5
Soybean meal (48%)	30.4
Fish meal (64.5%)	5.6
Soybean oil	5.9
Calcium hydrophosphate	1.2
Limestone	1.0
Salt	0.2
DL-methionine	0.2
Premix ^1^	1.0
Approximate composition of the test diets ^2^	
Crude protein	23.00
Metabolisable energy, (MJ/kg)	13.38
Lysine	1.40
Methionine	0.58
Methionine + cysteine	0.94
Calcium	1.02
Available phosphorus	0.47

^1^ The approximate composition provides the following per-kg diet: Vitamin A, 13800 IU/kg; vitamin D, 3600 IU; vitamin E, 24 IU/kg; vitamin K_3_, 3.6 mg; vitamin B_1_, 1.5 mg; vitamin B_2_, 6.6 mg; vitamin B_6_, 3 mg; vitamin B_12_, 0.015 mg; folate, 0.9 mg; biotin, 0.09 mg; D-pantothenic acid, 9.6 mg; nicotinamide, 36 mg; iron, 96 mg; zinc, 53.9 mg; manganese, 71.4 mg; copper, 12 mg; selenium, 0.3 mg; iodine, 0.42 mg. ^2^ Calculated value.
